# Epidemiology of Ebolaviruses from an Etiological Perspective

**DOI:** 10.3390/pathogens12020248

**Published:** 2023-02-03

**Authors:** Sahil Jain, Svetlana Khaiboullina, Ekaterina Martynova, Sergey Morzunov, Manoj Baranwal

**Affiliations:** 1 Department of Biotechnology, Thapar Institute of Engineering and Technology, Patiala 147004, Punjab, India; 2 Department of Biochemistry and Molecular Biology, Faculty of Life Sciences, Tel-Aviv University, Tel-Aviv 6997801, Israel; 3 Institute of Fundamental Medicine and Biology, Kazan Federal University, 420008 Kazan, Tatarstan, Russia; 4 Department of Pathology, School of Medicine, University of Nevada, Reno, NV 89557, USA

**Keywords:** ebolavirus outbreaks, ebolavirus reservoirs, ebolavirus transmission, ebolavirus in Africa

## Abstract

Since the inception of the ebolavirus in 1976, 32 outbreaks have resulted in nearly 15,350 deaths in more than ten countries of the African continent. In the last decade, the largest (2013–2016) and second largest (2018–2020) ebolavirus outbreaks have occurred in West Africa (mainly Guinea, Liberia, and Sierra Leone) and the Democratic Republic of the Congo, respectively. The 2013–2016 outbreak indicated an alarming geographical spread of the virus and was the first to qualify as an epidemic. Hence, it is imperative to halt ebolavirus progression and develop effective countermeasures. Despite several research efforts, ebolaviruses’ natural hosts and secondary reservoirs still elude the scientific world. The primary source responsible for infecting the index case is also unknown for most outbreaks. In this review, we summarize the history of ebolavirus outbreaks with a focus on etiology, natural hosts, zoonotic reservoirs, and transmission mechanisms. We also discuss the reasons why the African continent is the most affected region and identify steps to contain this virus.

## 1. Introduction

Since 1976, members of the *Ebolavirus* genus have been linked to multiple Ebola virus disease (EVD) outbreaks in Africa, characterized by diverse incidence rates and lethality [1]. Among the six known species of ebolaviruses, four can cause the hemorrhagic fever known as EVD [2]. In 1976, the Sudan virus (SUDV; sp: *Sudan ebolavirus*) was the first isolated during an outbreak in Nzara, Sudan [3]. In the same year, the Ebola virus (EBOV; sp: *Zaire ebolavirus*) was reported in the Yambuku outbreak, the Democratic Republic of the Congo (DRC) [4]. Since then, SUDV and EBOV have been linked to multiple outbreaks [1]. Nearly a decade later, the Reston virus (RESTV; sp: *Reston ebolavirus*) was isolated from Philippian crab-eating macaques (*Macaca fascicularis*) imported to a primate facility in Reston, Virginia [5,6]. Interestingly, RESTV is the only filovirus of Asiatic origin [7], as all previously identified viruses were from Africa [3,4,8]. RESTV can infect monkeys and pigs; however, no human cases have been reported due to this virus [9,10,11]. In 1994, the Taï Forest virus (TAFV; sp: *Taï Forest ebolavirus*) was isolated from an ethnologist who performed an autopsy on a dead chimpanzee [12]. Currently, this remains the only known TAFV case [1]. The fifth member, the Bundibugyo virus (BDBV; sp: *Bundibugyo ebolavirus*), was retrospectively named after an outbreak in Bundibugyo, Uganda, in 2007 [13]. BDBV was linked with two small outbreaks, and no cases have been reported since 2012 [1]. The sixth ebolavirus species was isolated in 2018 from *Mops condylurus* (Angolan free-tailed bat) and *Chaerephon pumilus* (little free-tailed bat) [14]. It was named the Bombali virus (BOMV; sp: *Bombali ebolavirus*). Along with RESTV, it is the second ebolavirus species believed to be non-symptomatic to humans, and no outbreaks have been linked to this virus.

EBOV infection has been linked to nearly two-thirds of ebolavirus outbreaks [1]. Since 2013, nine EBOV outbreaks have been documented, with seven located in the DRC, Central Africa [10]. The 2013–2016 West African outbreak, declared an epidemic, was characterized by the highest number of fatal cases, accounting for ~74% of all ebolavirus-caused deaths [1,15,16]. This epidemic also demonstrated the EBOV circulation in West Africa, indicating an expansion of the endemic area. EBOV has also been linked to the second biggest ebolavirus outbreak (2018–2020) in DRC, resulting in 2287 deaths [1,8]. As of 28 May 2022, 32 outbreaks with more than 34,750 EVD cases and 15,350 deaths (44.2%) were registered [1,17].

Multiple natural hosts and zoonotic reservoirs have been identified as plausible carriers of ebolaviruses [18]. EBOV antibodies have been found in mammals such as duikers (*Cephalophus dorsalis*); bats (frugivorous and insectivorous); dogs (*Canis familiaris*); pigs (*Sus scrofa*); and non-human primates (NHPs) such as drills (*Mandrillus leucophaeus*), mandrills (*Mandrillus sphinx*), chimpanzees (*Pan troglodytes*), gorillas (*Gorilla gorilla*), and baboons (*Papio Anubis*) [18,19,20]. Though serologically unconfirmed, some rodents [7] and arthropods, especially bed bugs, have been suggested as hosts for ebolaviruses [21,22]. The natural reservoir appears to have intermittent contact with dead-end hosts, such as humans, duikers, and NHPs [23]. Though bats have been primarily suggested as natural hosts, there is still definitive evidence missing to confirm this.

The source of infection has often remained unidentified for most of the outbreaks. Ebolaviruses can transmit via oral, respiratory mucosal, conjunctival, and submucosal routes in NHPs [24,25]. Activities such as hunting, bushmeat trading, consuming contaminated fruits, or contacting bat, porcupine, or antelope carcasses are suggested as modes of virus transmission [26,27]. After infection, the virus can spread via person-to-person transmission upon direct contact with body fluids such as stools, urine, sweat, tears, breast milk, and saliva or by contact with damaged skin [28,29]. In addition, sexual intercourse with a male survivor can transmit the virus, as it can be found in semen for months after recovery [30]. Traditional funeral rituals, including contact with a corpse, can also contribute to virus transmission [31]. Exponential nosocomial transmission has been shown in hospital staff and healthcare workers (HCWs) upon using unautoclaved needles and instruments. HCWs have become infected when treating patients outside of designated areas and without using personal protective equipment (PPE) [27].

This review discusses ebolavirus outbreaks, focusing on the viral etiology. We consider the potential natural hosts of ebolaviruses and the modes of transmission. Additionally, we analyzed the basis for ebolavirus outbreaks to be restricted to Africa, and discuss measures that could prevent potential ebolavirus epidemics and pandemics.

## 2. Epidemiology

### 2.1. Democratic Republic of the Congo

**Figure 1:**
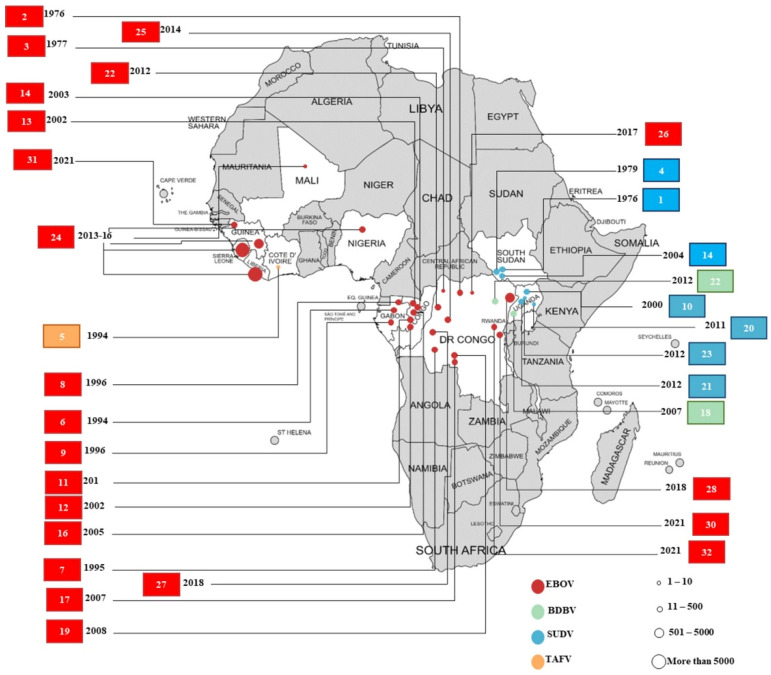
All ebolavirus outbreaks registered to date. The size of the circle represents the number of cases, while the color of the circle represents the ebolavirus type responsible for the outbreak. The squares indicate the chronological order of the outbreak, while the color of the squares corresponds to the ebolavirus type.

The first EBOV outbreak was recorded in the DRC (formerly Zaire) on 26 August 1976, when a male faculty member at a mission school in Yambuku village was reported to be EBOV-positive after eating bushmeat [3,4]. Nosocomial transmission is thought to have been the most powerful amplifier in this outbreak. Three hundred and eighteen cases were reported by 24 October 1976, with a fatality rate of 88% [4,32] (Table 1). In June 1977, a second EBOV outbreak was reported in Tandala, DRC (Figure 1), resulting in a single fatal case of a nine-year-old girl [33]. A retrospective analysis suggested a few more unconfirmed cases during this outbreak [33]. The source of infection for this outbreak could not be identified [33].

The third EBOV outbreak in the DRC was documented on 6 January 1995 [34]. It is believed that a few patients hospitalized in April 1995 were initially misdiagnosed as having dysentery and typhoid, which led to the nosocomial transmission of EBOV infection [34]. Retrospective studies have indicated a charcoal maker in Kikwit, DRC as the index case, though the source of infection remains unknown [1,34]. Overall, 315 cases were reported with a mortality rate of 79.37%, and the last death was registered on 16 July 1995 [34].

The fourth DRC outbreak commenced in the Kasai-Occidental province (Figure 1) in May 2007 and was officially recognized in September [1,26]. It was the first EVD incidence where bats (*Hypsignathus monstrous* and *Epomops franqueti*) were suspected as the primary source of infection [26]. Supporting this suggestion was the fact that the local animal population was not infected during the outbreak [26]. In addition, the Kasai-Occidental province has a limited NHP population, reducing the probability of viral transmission by contact with an infected animal [26]. Moreover, index cases reportedly purchased bats from hunters [26]. Traditional burial practices were the main amplifiers of this outbreak, which ended on 20 November 2007 [1,26]. In December 2008, a small EBOV outbreak was documented in the DRC [1], which ended on 16 February 2009 with a mortality rate of 47% [35] (Table 1).

**Table 1:** All human Ebola virus (EBOV) outbreaks since 1976. We present the number of cases and deaths recorded by the CDC where possible. However, it should be noted that the actual number of confirmed cases might vary, as suggested by the ambiguity in various reports and published literature. This might affect the % mortality mentioned in the table.

Year	Country	% Mortality (Deaths/Cases)	Reference	Year	Country	% Mortality (Deaths/Cases)	Reference
Ebola Virus (before 2014)				Ebola Virus (2014 onwards)			
1976	DRC *	88.05 (280/318)	[1,32]	2013–2016	Guinea	66.72 (2543/3811)	[15]
1977	DRC	100 (1/1)	[1,33]		Liberia	45.04 (4810/10,678)	[15]
1994	Gabon	59.62 (31/52)	[1,31]		Sierra Leone	28 (3956/14,124)	[15]
1995	DRC	79.37 (250/315)	[1,34]		Mali	75 (6/8)	[36]
1996	Gabon	67.74 (21/31)	[1,31]		Nigeria	40 (8/20)	[36]
1996 ***	Gabon	75 (45/60)	[1,31]		Senegal	0 (0/1)	[36]
2001	Gabon/RC **	81.54 (53/65)	[1,37]	2014 ***	DRC	71.01 (49/69)	[1,38]
2002	Gabon/RC	74.58 (44/59)	[1]	2017	DRC	50 (4/8)	[1,39,40]
2002 ***	RC	89.51 (128/143)	[1,41]	2018 ***	DRC	61.11 (33/54)	[1]
2003	RC	82.86 (29/35)	[1,42,43]	2018	DRC	65.91 (2287/3470)	[1,8]
2005	RC	83.33 (10/12)	[1]	2020	DRC	42.31 (55/130)	[1]
2007	DRC	70.83 (187/264)	[1,26]	2021	DRC	50 (6/12)	[1,44]
2008	DRC	46.88 (15/32)	[1]	2021	Guinea	52.17 (12/23)	[1,10,45]
				2021 ***	DRC	54.55 (6/11)	[1,44]

* DRC stands for the Democratic Republic of the Congo. ** RC stands for the Republic of the Congo. *** These outbreaks differed from the other outbreaks witnessed in the same year.

A BDBV outbreak was reported in the DRC on 17 August 2012 [46]. It was the first registered ebolavirus outbreak in the DRC caused by a species other than EBOV. An HCW in the Orientale province presented with EVD symptoms on 28 June 2012 [47]. A definitive source of infection could not be established, as the HCW acknowledged contact with bats, had attended funerals, and also had direct contact with sick people [47]. The outbreak lasted 102 days, ending on 26 November 2012, with a fatality rate of 34.21% [48] (Table 2).

Nearly two years later, an EBOV outbreak was documented in Inkanamongo village, DRC on 26 July 2014 [38]. This outbreak was concurrent with the 2013–2016 West African EBOV epidemic (discussed later) [38]. A pregnant woman was in contact with a dead monkey and was identified as the index case [38]. Physical contact with the victim and contact with her body fluids were the main infection amplifiers [38]. No cases were reported after 7 October 2014 [38]. The next DRC outbreak was reported in the Likati district (Figure 1) in March 2017 [39,40]. The index case frequently bought fish, ate cooked *Potamochoerus porcus* (red river hog) two weeks prior to the symptom onset, and bought raw *Eidolon helvum* a week before the symptoms began [39]. Therefore, the primary source of this outbreak could not be definitively identified. The outbreak ended on 2 July 2017, with a fatality rate of 50% [39].

The largest DRC outbreak started in May 2018 in Ituri and North Kivu, DRC [1,49], and it was officially declared on 1 August 2018 [50]. It is recognized as the second largest ebolavirus outbreak [1] and was announced as a “public health emergency of international concern” (PHEIC) by the World Health Organization (WHO) on 17 July 2019 [51]. The primary transmission source and the index case remain unknown [8]. Traditional burial practices, violent attacks on HCWs, and general community mistrust towards “Western” treatments were cited as major contributors to the propagation of the infection [8]. The outbreak ended on 25 June 2020 [52]. At the same time, a small outbreak in Bikoro, DRC was reported on 8 May 2018, which ended on 24 July 2018 [1]. It was the first outbreak during which an Ebola vaccination campaign was initiated [53]. During the last stages of the outbreak in Ituri and North Kivu, a separate outbreak was documented in Mbandaka, DRC on 1 June 2020, which ended on 18 November 2020 [1]. These two concurrent outbreaks accounted for 184 cases and 88 deaths (Table 1). The primary source of both outbreaks is still unknown.

**Table 2:** All ebolavirus outbreaks caused by viruses other than EBOV since 1976. We present the number of cases and deaths recorded by the CDC where possible. However, it should be noted that the actual number of confirmed cases might vary, as suggested by the ambiguity in various reports and published literature. This might affect the % mortality mentioned in the table.

Year	Country	% Mortality (Deaths/Cases)	Reference
SUDV			
1976	South Sudan	53.17 (151/284)	[4]
1979	South Sudan	64.71 (22/34)	[54]
2000	Uganda	52.71 (224/425)	[55]
2004	South Sudan	41.18 (7/17)	[1]
2011	Uganda	100 (1/1)	[56]
2012	Uganda	36.36 (4/11)	[57]
2012	Uganda	50 (3/6)	[57]
**TAFV**			
1994	Ivory Coast	0 (0/1)	[12]
**BDBV**			
2007	Uganda	32.06 (42/131)	[1,13]
2012	DRC	34.21 (13/38)	[48]

Two small EBOV outbreaks were recorded in North Kivu, DRC on 7 February 2021 and 8 October 2021, claiming 12 lives [1,44]. These outbreaks ended on 3 May 2021 and 16 December 2021, respectively [1,44]. Both outbreaks were thought to be initiated by either a disease relapse or sexual intercourse with a survivor [10,45,58].

### 2.2. Gabon and the Republic of the Congo

In 1994, an EBOV outbreak was reported for the first time in Gabon (Figure 1), making it the second endemic Central African country after the DRC. This outbreak started in Makakou in December 1994 and ended on 17 February 1995 [31] (Table 1). There were 52 cases reported with a mortality rate of 59.62% [31]. The index patients were identified as gold-panning workers near a rainforest [31], suggesting contact with an EBOV zoonotic reservoir as the potential source of infection. Two EBOV outbreaks were reported in Gabon in the following year, presumably caused by bushmeat consumption [31]. The first outbreak was in Mayibout 2, Gabon in February 1996, while the second was in the Booué town on 5 October 1996 [31]. There were a total of 66 fatal cases reported (Table 1).

The eleventh ebolavirus and seventh EBOV outbreak started on the border of Gabon and the Republic of the Congo (RC) on 25 October 2001 [37]. It was declared on 11 December 2001 and was the first documented outbreak in the RC [37]. This was also the first case of an ebolavirus outbreak that spread across country borders, wherein 53% and 47% of the cases were reported in Gabon and the RC, respectively [37]. The primary mode of transmission was thought to be due to bushmeat hunting, with index cases having contact with NHPs, which were retrospectively found to be infected with EBOV [37]. This outbreak ended on 6 May 2002 [37]. In less than two weeks, a hunter reportedly had EVD-like symptoms after handling a dead chimpanzee [37]. Later, on June 2002, another outbreak was confirmed in Gabon and the RC, claiming 44 lives [1]. A third consecutive outbreak in the RC was documented in November 2002, wherein EBOV infection was diagnosed in the Kéllé district [59]. Once again, the suspected source of infection was NHPs [60]. This outbreak ended in June 2003 with 143 cases [41,61]. There were 128 (89.51%) fatal cases [41,61], which represents the highest mortality rate of all the ebolavirus outbreaks recorded in the RC to date (Table 1).

Next, in November 2003, an EBOV outbreak was documented in the Mbomo district, RC (Figure 1), where monkey meat was suspected to be the source of transmission [41,42,43]. Thirty-five cases were reported in this outbreak, with 29 fatalities (Table 1) [41,42,43]. The fifth consecutive EBOV outbreak in the RC was reported on 18 April 2005; two hunters were infected in the Etoumbi district after consuming an unidentified quarry [1,59]. Nosocomial transmission and traditional burial practices were identified as propagating factors for infection, though no HCWs were infected in this outbreak [59]. The outbreak ended on 8 July 2005 [59]. No more outbreaks have been registered in the RC to date.

### 2.3. Uganda

Ebolaviruses first appeared in Uganda (East Africa) on 30 August 2000, when the index case of the largest SUDV outbreak was documented in the Gulu district [62]. The outbreak was reported as an ebolavirus outbreak on 14 October 2000, and spread to Uganda’s Masindi and Mbarara districts [62]. It was one of the first outbreaks in which an ebolavirus was reported to cause abortions in pregnant women [55]. In addition, it was perhaps the first instance where traditional burial practices were reported as the main reason for the spread of infection [62]. There were 425 cases (393, 5, and 27 in Gulu, Masindi, and Mbarara, respectively) and 224 deaths (203, 4, and 17 in Gulu, Masindi, and Mbarara, respectively) during this outbreak, which ended on 27 February 2001 [55,63].

The first ever BDBV outbreak was reported in August 2007 in the Bundibugyo district, Uganda, where 131 people were infected, resulting in a mortality rate of 32% [1,13] (Table 2). The outbreak was announced on 29 November 2007 and ended on 20 February 2008. The index case was identified as a 26-year-old pregnant woman who was suspected of being infected while hunting [64].

On 1 May 2011, an isolated fatal case of an SUDV-infected girl was documented in Luwero, Uganda [56]. The source of this outbreak has not been identified, though multiple bat species were reported to roost inside the school the girl was attending [56]. In 2012, two small SUDV outbreaks were confirmed in Uganda on 26 July and 13 November, respectively, with seven fatal cases documented [57]. The WHO, CDC, and DRC Ministry of Health (DRCMoH) measures contained these outbreaks, which ended on 4 October 2012 and 16 January 2013, respectively [57]. The primary source of infection remains unknown for both outbreaks [57].

### 2.4. South Sudan

SUDV infection was diagnosed for the first time on 27 June 1976 in South Sudan [3,8]. It was also the first documented human ebolavirus infection [3,8]. In this outbreak, an employee in a cotton factory in Nzara, South Sudan (Figure 1) was identified as the index case [3,8]. This patient was the source of infection for other workers, relatives, and HCWs [3]. A total of 284 people were infected and 151 of them died [4] (Table 2), with the last case reported on 25 November 1976 [3]. The second SUDV outbreak was reported three years later on 31 July 1979 in South Sudan [3]. Twenty-two patients died (Table 2) before the outbreak ended on 6 October 1979. A retrospective analysis indicated that the infected patients were directly linked to the index case, and the virus was suggested to lose its severity upon human-to-human transmission [54]. The source of infection remains unknown for both the 1976 and 1979 SUDV outbreaks [3].

In 2004, simultaneous SUDV and measles outbreaks were reported in Yambio County, South Sudan [1]. Seven deaths were attributed to EVD infection [1] (Table 2). Information about the primary source or index case remains unknown. It was the last incidence recorded in South Sudan to date.

### 2.5. West African Countries

The longest-lasting, most widespread ebolavirus outbreak with the highest morbidity and mortality rates was the EBOV epidemic recorded in 2013–2016, West Africa. It started in December 2013, though the causative EBOV was identified later, on 21 March 2014 [36,65]. It was the first time an EBOV was isolated from West Africa. In this epidemic, 28,652 cases were diagnosed with 11,325 deaths (39.65%) in various West African countries, such as Guinea, Liberia, and Sierra Leone, before it ended [15] (Table 1). Eight hundred and ninety-eight HCWs reported EVD symptoms, of which 518 died [15]. The index case was identified as a two-year-old Guinean boy playing near trees that were the roosting site for bats [66]. The epidemic was announced by the WHO as a PHEIC from 8 August 2014 to 29 March 2016, and it was the first outbreak ever to be classified by the United Nations Security Council as a “threat to international peace and security” [15,16,67]. The epidemic ended on 9 June 2016 [68].

A small EBOV outbreak was registered in Guinea on 14 February 2021 [10,45]. It was suggested that it started by contact with a 2013–2016 epidemic survivor. There were 12 fatal cases documented before the end of the outbreak on 19 June 2021 [1]. The index case was identified as an HCW [45].

### 2.6. Other Outbreaks and Incidents

In 1994, an ethnologist was diagnosed with a TAFV infection while carrying out a chimpanzee autopsy in Côte d’Ivoire [12]. Interestingly, this was the first direct ebolavirus transmission from an infected NHP to a human. This is also the only case reported as having been caused by TAFV infection to date.

Some incidences, rather than outbreaks, have been recorded outside of endemic regions. In 1976, a British investigator was accidentally self-inoculated while processing patient samples [69]. The patient survived, and no more cases of EBOV exposure were reported [69]. In 1996, a South African HCW volunteer in Gabon felt ill and returned to his homeland [1,70]. Though he survived, a nurse in charge of this patient was diagnosed with EVD and eventually died [1]. In 1996 and 2004, two fatal cases were reported involving Russian scientists working with laboratory strains of ebolaviruses [71,72]. During the 2013–2016 epidemic, an Italian and a British HCW returning to their respective countries after voluntarily serving in Sierra Leone developed EVD symptoms post-arrival [1]. These patients survived, and no other cases linked to these cases were reported [1]. During the same outbreak, a patient from Sierra Leone receiving treatment in Spain was the source of an HCW infection [1,73]. While the HCW recovered, the patient died, and no more cases were reported [1]. Some U.S. HCW volunteers in Africa were reported to develop EVD symptoms. Additionally, some African patients receiving treatment in the U.S. were the source of the EBOV infection of U.S. HCWs [36]. Though two of the patients died, all HCWs recovered [36].

## 3. Etiology of Ebolavirus Infections

**Figure 2:**
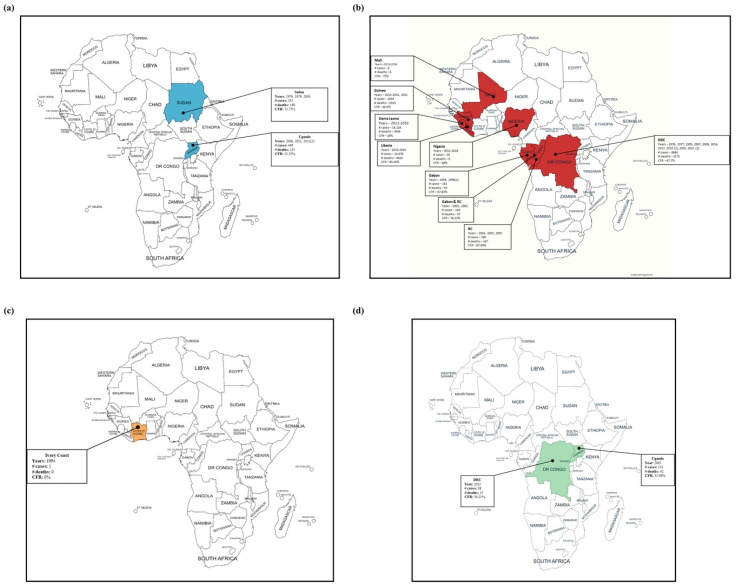
A map presenting African countries affected by specific ebolaviruses. The date of an outbreak, number of cases, deaths, and CFR per country are presented. (**a**–**d**) represent areas affected by SUDV, EBOV, TAFV, and BDBV, respectively. We present the number of cases and deaths recorded by the CDC where possible. However, it should be noted that the actual number of confirmed cases might vary, as suggested by the ambiguity in various reports and published literature. This might affect the CFR mentioned in the figure.

The spread of ebolavirus infections has been documented mainly in African countries near the equator, where the landscape has dense forests and vegetation [21]. Three of the thirty-two ebolavirus outbreaks were registered in each of Northeast Africa and West Africa, while twenty-one and five were registered in Central Africa and East Africa, respectively (Figure 2). Approximately 41% of outbreaks were in DRC, with six EBOV outbreaks reported in the last five years (Figure 2). Twenty-two (twenty in Central Africa, two in West Africa), seven (three in Northeast Africa, four in East Africa), one (in West Africa), and two (one in East Africa, one in Central Africa) outbreaks were caused by EBOV, SUDV, TAFV, and BDBV, respectively (Table 3). It appears that most of these outbreaks, including the 2013–2016 epidemic and 2018–2020 outbreak, were caused by EBOV. Central African countries seem to be the most active sites of EVD (Figure 2). Uganda and South Sudan (East African countries) have never recorded an EBOV outbreak, while fatal outbreaks in West African countries seem to be caused solely by EBOV (Table 3). In addition, SUDV outbreaks have never been reported outside the East African region (Figure 2, Table 3). It should be noted that the two ebolavirus outbreaks with the maximum number of cases (2013–2016 epidemic and 2018–2020 outbreak) were recorded in the last decade. This indicates a possible evolutionary adaptation of the virus, especially for the *Zaire ebolavirus* species, which may have consequently led to its increased virulence.

An analysis of the ebolavirus outbreak history suggests that the DRC (13), Gabon/the RC (8), and Uganda (5) are the most active foci where the majority of outbreaks have been documented. These are neighboring countries, suggesting that the migration of the natural ebolavirus hosts contributes to the frequent outbreaks observed there. In addition, EBOV seems restricted to Central and Western Africa, suggesting it may thrive in humid rainforests, which are abundant in these regions [74]. It is intriguing to note that most of the EBOV outbreaks have been documented in the dry season, while the SUDV outbreaks were registered in the wet season [23]. This observation suggests the presence of multiple natural reservoirs of ebolaviruses, which are active in different climatic conditions [23]. In some countries, such as Gabon, the dry season is also the fruiting season [75]. This suggests that multiple animals (intermediate or dead-end hosts) feeding around the same regions may come into contact with natural reservoirs, consequently initiating the transmission chain. It should be noted that the 2013–2016 epidemic indicated EBOV circulation in West Africa. The timing of this outbreak coincided with the dry, fruiting season, suggesting a spillover of viruses from natural hosts to other animals during feeding time [18].

The natural host and source of infection for many of these outbreaks remain unknown. Therefore, it is crucial to assess the etiology of ebolavirus outbreaks to develop appropriate protection and prevention measures. Different sources of infection have been suggested for various ebolavirus outbreaks. Four (2007 in the DRC, 2011 in Uganda, 2012 in the DRC, and 2013–2016 in West Africa), two (1976 in the DRC and 1996 in Gabon_b_), seven (1994 on the Ivory Coast, 1996 in Gabon_a_, 2001 in Gabon/the RC, 2002 in Gabon/the RC, 2002 in the RC, 2003 in the RC, and 2014 in the DRC), and three (2021 in the DRC, 2021 in Guinea, and 2021 in the DRC_b,_ a and b indicate the chronology of the outbreaks which occurred in the same year.) outbreaks have been linked to contact with bats, bushmeat consumption, NHPs, and infection relapse, respectively. Still, little information regarding the source of infection is available for several outbreaks.

### 3.1. Bats as the Source of Infection

Interestingly, it has been suggested that bat exposure caused at least one each of the EBOV, SUDV, and BDBV outbreaks. The 2007 DRC EBOV outbreak in the Kasai-Occidental province is believed to have been initiated by contact with infected bats [1]. It could be suggested that close contact with dead bats and their blood is the mechanism of virus transmission. Two bat species, *H. monstrosus* and *E. franqueti*, that were detected as positive for EBOV RNA [76] were identified amongst the migratory bat populations in the DRC [26]. In the 2011 SUDV case, in which a 12-year-old girl was infected in Uganda, family members confirmed she was not exposed to any dead animals, did not visit sick relatives, and had not been to any funerals [56]. Further investigations found that bats, including the *Epomophorus*, *Hipposideros*, *Pipistrellus*, and *Chaerophon* species, roosted in the school the girl attended [56]. This suggests that she was exposed to SUDV-infected bats [56]. However, if this was the case, it is unclear as to why other school children and staff were not infected.

In the 2012 DRC BDBV outbreak, the index case was identified as a nurse who had contact with sick patients, had attended funerals, and had contact with bats [46]. Although multiple sources of exposure could be involved in initiating the infection, contact with bats could not be ruled out as the single source of infection. In the 2013–2016 West Africa outbreak, the index case was linked to direct contact with *M. condylurus*, an insectivorous bat species [18]. The two-year-old index case was retrospectively found frequently playing around trees that served as a roosting site for *M. chondylurus* [77]. This theory was supported by the fact that no hunters were enlisted in the first few cases, which ruled out bushmeat or NHP carcasses as the initial sources of viral transmission [77]. Moreover, no decline in the population of the susceptible animals, concurrent with or prior to the outbreak, was reported [77], supporting the bat-origin hypothesis. Interestingly, *M. chondylurus* also roosts in highly human-populated areas. It could be suggested that if *M. chondylurus* is a primary source of EVD infection, then many other outbreaks in highly populated areas should be registered. *M. chondylurus* was not found at the sites of many outbreaks [78], suggesting that these bats are not the primary source of infection or that there are multiple sources of infection at different outbreak sites. Therefore, further investigations are needed to confirm the role of bats in these outbreaks.

### 3.2. Bushmeat and NHPs as the Source of Infection

Bushmeat consumption has been suggested as the source of infection in the 1976 EBOV outbreak, when a 44-year-old male was diagnosed with EVD in Yambuku, DRC [3,4]. During the first 1996 Gabon outbreak, 18 people processed chimpanzee meat before having symptoms of EVD [31]. In the same year, a 39-year-old hunter came in contact with a dead chimpanzee in Booué, starting the second 1996 Gabon outbreak [31]. From 2001 to 2003, contact with or the consumption of duiker, chimpanzee, monkey, or gorilla carcasses was identified as the primary cause of four consecutive RC EBOV outbreaks [41,42,43,60]. Later, in the 2014 DRC outbreak, a pregnant woman who had contact with dead monkey meat was retrospectively identified as the index case [38]. However, NHPs may not be considered the primary reservoir for ebolaviruses, as they are susceptible to infection and act as dead-end hosts [23]. In addition, many NHPs were killed during the 1994–2005 outbreaks [61,79]. Therefore, the source of NHP infection remains unclear.

It appears that the natural hosts of ebolaviruses remain elusive. To effectively control potential EVD epidemics and pandemics, it is crucial to identify the primary hosts, amplifying hosts, and zoonotic reservoirs of ebolaviruses. A chain of transmission also needs to be established to better target the underlying infection mechanisms.

## 4. Potential Reservoirs

### 4.1. Bats

**Figure 3:**
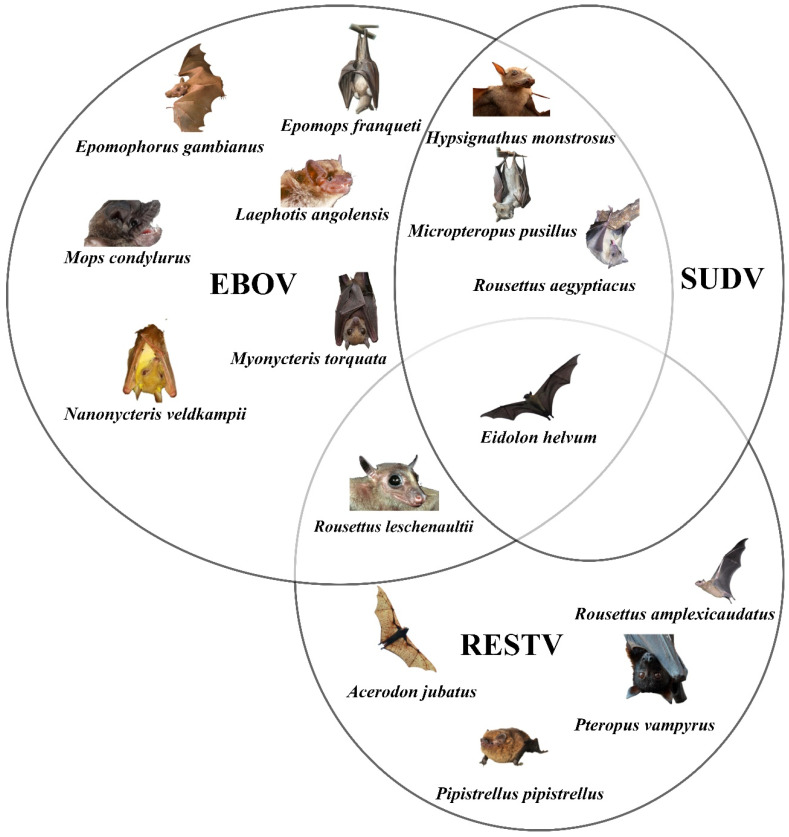
A Venn diagram presenting the bat species found to be positive for ebolavirus antibodies. *Eidolon helvum* and *Rousettus leschenaultii* species have been detected to possess EBOV, SUDV, and RESTV antibodies.

Bats have been suggested to be the most probable natural reservoirs for ebolaviruses. More than 1400 bat species (residential and migratory) have been tracked in Africa, and nearly 100 species could be potential reservoirs for filoviruses [19,80]. In 2005, the *Hypsignathus monstrosus* (hammer-headed fruit bat), *Epomops franqueti* (Franquet’s epauletted fruit bat), and *Myonycteris torquata* (little collared fruit bat) bat species, collected from the 2001 and 2003 outbreaks sites, were shown to have asymptomatic infections through the detection of ebolavirus RNA in their livers and spleens [76]. Later, in 2007, EBOV-specific antibodies were found in these three bat species [81]. EBOV-specific antibodies have also been detected in the *Eidolon helvum* (straw-colored fruit bat), *Epomophorus gambianus* (Gambian epauletted fruit bat), *Laephotis angolensis* (Angolan long-eared bat), *Micropteropus pusillus* (Peters’s dwarf epauletted fruit bat), *Mops condylurus* (Angolan free-tailed bat), *Nanonycteris veldkampii* (Veldkamp’s dwarf epauletted fruit bat), *Rousettus aegyptiacus* (Egyptian fruit bat), and *Rousettus leschenaultii* (Leschenault’s rousette) bat species [81,82,83,84,85,86,87] (Figure 3). Additionally, *H. monstrosus*, *M. pusillus*, and *R. aegyptiacus* were found to be positive for SUDV-specific antibodies [87], while *Acerodon jubatus* (golden-capped fruit bat), *Pipistrellus pipistrellus* (common pipistrelles), *Pteropus vampyrus* (large flying fox), *Rousettus amplexicaudatus* (Geoffroy’s rosettes), and *R. leschenaultii* had RESTV-specific antibodies [86,88,89,90] (Figure 3). *E. helvum* has been reported to have antibodies against all the ebolavirus species that are pathogenic to humans (EBOV, SUDV, TAFV, and BDBV) as well as RESTV [91] (Figure 3). Several studies have demonstrated BOMV-specific antibodies in some insectivorous bat species [14,92,93]. These data support the hypothesis that bats are a natural reservoir for ebolaviruses [19,81].

It is an intriguing idea that bats are the prime reservoir candidates. Bats can be hosts for several viruses concurrently, without a symptomatic infection [94,95]. This could be explained by an unusual reaction of the immune system to viral infection, which supports a viral presence [18]. The specific, adaptive evolution of various immune and metabolic genes, such as pattern recognition receptor-encoding genes and mitochondrial oxidative phosphorylation genes, have uniquely tailored the bat defense and metabolic systems, enabling them to tolerate and support various viruses [80]. The modulation of initial viral replication, the suppression of viral replication during flight, and a limited inflammatory response to viruses are some other characteristics enabling bats to host viruses [94,95,96]. Therefore, bats can carry and potentially transmit viruses in a sizeable geographic area. Also supporting their role as ebolavirus reservoirs are reports of large bat populations in some endemic regions, which could contribute to frequent interactions with secondary or dead-end hosts, resulting in viral transmission [95].

Despite considerable evidence suggesting bats as an ebolavirus reservoir, there are data that this hypothesis does not explain. For example, no ebolavirus outbreaks have been registered in Equatorial Africa, despite the large bat population and their use as bushmeat [26,97]. In addition, bat hunters have never been the index case for any Ebola virus outbreaks [18,98]. Additionally, ebolavirus-specific antibodies have been found in bats roosting in non-endemic areas, which is puzzling. In contrast, there was no evidence of ebolavirus infection in the bats of nearly 40 bat species in endemic areas [87].

It should be noted that the detection of ebolavirus-specific antibodies could be an indicator of an earlier infection [87,99,100]. Therefore, the detection of viral RNA or the isolation of the virus from bats could be evidence of bats being ebolavirus reservoirs. Currently, the work published by Leroy et al. (2005) is the only study demonstrating the presence of EBOV RNA in tissue samples of three (*H. monstrosus*, *E. franqueti*, and *M. torquata*) bat species obtained from Gabon [76]. In another study, the RNA of the non-pathogenic human RESTV was detected in *Miniopterus schreibersii* (Schreibers’s long-fingered bat) tissue from the Philippines [90]. However, most reports have indicated only the presence of ebolavirus-specific antibodies [44,77,78,86,87,91]. These data suggest that bats may be the natural reservoirs; however, the demonstration of viral RNA or the isolation of ebolaviruses from bat tissue remains essential to support this hypothesis.

### 4.2. Other Potential Reservoirs

Some other mammals have also been suggested to be natural reservoirs of ebolaviruses. A study from 1999 reported finding EBOV RNA in murids (rodents) and shrews [101]. In another investigation, a serum sample from *Anomalurus derbianus* (Lord Derby’s scaly-tailed squirrel) captured from the DRC exhibited reactivity to EBOV antigens via an immunofluorescent assay (IFA). However, no sign of antibody presence was able to be confirmed by a radioimmunoassay [102]. In another study, the presence of EBOV RNA fragments was demonstrated in tissue samples of the *Mus setulosus* (Peter’s mouse), *Sylvisorex ollula* (the greater forest shrew), and *Praomys* species [78,101]. Pigs have also been suggested as a potential reservoir. This assumption is based on the detection of RESTV in a Philippian *Sus scrofa domesticus* (domestic pig) co-infected with porcine reproductive and respiratory syndrome virus (PRRSV) [11]. The pig-to-human transmission of RESTV infection was also contemplated, based on finding anti-RESTV antibodies in pig farm workers [11]. In 2014, a similar report from Shanghai, China indicated RESTV infection in piglets co-infected with PRRSV [103]. Later, in 2016, the first report of EBOV-specific antibody detection in domestic pigs from Africa was presented [104]. However, an epidemiology analysis was not able to support the role of pigs in any ebolavirus outbreaks.

Arthropods have also been suggested to play a role as natural reservoirs or in ebolavirus maintenance. Bennett et al. found non-ebolavirus RNAs in arthropods (fig wasps and primitive crane flies) that were ecologically linked to bat habitats [105]. In addition, Dutto et al. suggested a role of arthropods role as ebolavirus vectors [106]. Based on these data, it could be suggested that arthropods play a role in the virus transmission to bats, thus representing the primary natural reservoir [105]. However, there is limited evidence linking EBOV transmission from arthropods to bats or other species. Leendertz suggested another alternative hypothesis in 2016, where aquatic and semi-aquatic bat prey were contemplated as the prime natural reservoir [107]. More studies are needed to confirm the role of these organisms in ebolavirus maintenance in nature.

## 5. Transmission

### 5.1. Reservoir-to-Animal Transmission

**Figure 4:**
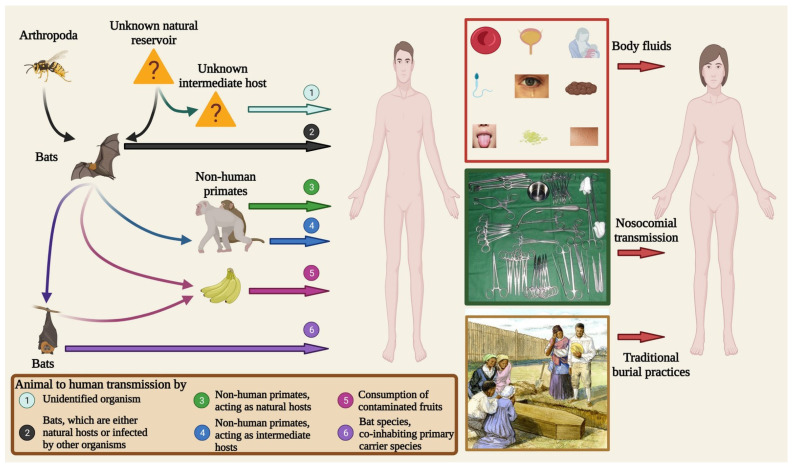
Plausible ebolavirus hosts and reservoirs, as well as modes of virus transmission. Bats are considered the primary carriers of ebolaviruses. Some virus transmission to bats may occur from arthropods. Bats could transmit the virus directly to humans or to other bats and NHPs, which serve as amplifying hosts before transmitting to humans. In addition, consuming fruits contaminated with bat saliva or droppings is a plausible mode of virus transmission. Direct infection from NHPs is also suggested. Human-to-human transmission could happen via three mechanisms: (a) contact with body fluids or secretions from a victim; (b) hospital settings, wherein the use of unsterilized needles or syringes and the non-availability of proper PPE equipment results in a brisk viral spread; and (c) traditional burial practices, which can involve close contact with a dead patient.

Natural reservoirs can transmit the virus to secondary, amplifying, and/or dead-end hosts [61,108] (Figure 4). There are several modes of transmission: the consumption of contaminated food, the consumption of a primary carrier, exposure to contaminated excreta, or co-inhabiting in the same regions. The consumption of fruits contaminated after exposure to infected bat saliva could cause virus transmission [109,110]. NHPs, such as monkeys and bonobos, might be infected upon consuming infected bats [111,112]. Bats could release viruses via excreta over large areas during flights [10]. Further, the transmission of the ebolavirus to secondary host bats that co-habitat with the primary hosts was also suggested by Shapiro et al. [113] (Figure 4). According to this hypothesis, the primary bat-carriers could transmit the virus to secondary bat-carriers, which then continue the transmission chain.

Additionally, animal-to-animal transmission is possible when infected prey is consumed by a susceptible animal [108]. The secondary host could serve as an additional reservoir or amplifying host. These secondary host animals could then transmit the virus to humans.

### 5.2. Reservoir/Secondary Host-to-Human Transmission

Human–bat interactions have been suggested as the source of infection in some outbreaks [26,77]. Bats are a common source of nutrition in Africa [114]. Therefore, hunting, butchering, and selling bat bounty in the local markets is common [18]. Additionally, the potential contacts increase due to some bat species being known to roost in houses and tunnels [115,116]. These data imply high-frequency human–bat contact, which could lead to virus transmission. Direct contact with yet unknown reservoirs could cause human virus transmission.

An analysis of multiple ebolavirus outbreaks indicated that their respective index cases had contact with dead or infected wildlife habituating local forests, caves, and mines [12,117,118,119]. The consumption of dead NHP meat is typical in Africa and, in some cases, could be the route of transmission [61] (Figure 4). Bush fires and droughts force animals’ migration, bringing them in closer contact with humans and thus increasing the chance of virus transmission. Human practices such as deforestation and agricultural activities cause habitat loss, increasing the potential for human–animal contact [19,120]. Additionally, the wildlife trade is a significant source of income for the native population, presenting a high risk of exposure to infected animals.

### 5.3. Human-to-Human Transmission

Ebolaviruses appear to spread between humans via oral contact, aerosol inhalation, fomites, and direct contact [10]. Contact with a patient’s blood, urine, feces, saliva, sputum, sweat, tears, breast milk, semen, vaginal fluid, or vomit could lead to virus transmission [121,122,123] (Figure 4). Reusing contaminated needles or razor blades could also result in the passing of the virus between individuals [124,125]. Fomite transmission could be mediated by contaminated instruments, door knobs, or restrooms [19]. It should be noted that nosocomial transmission is common and accounts for a high percentage of EBOV morbidity and mortality [126].

## 6. Ebolavirus: The African Healthcare Threat

Ebolaviruses have historically been isolated to Africa, and especially to equatorial countries. Several factors could explain these reports. Most of the African population resides in highly bat-populated areas [95], suggesting a high risk of contact with infected mammals. Essential practices, such as hunting local animals for trade and the consumption of bushmeat, increases the risk of virus exposure in Africa [127,128]. Catching bats is practiced from childhood, with bat-inhabited caves being shelters and bat-bitten fruits being used as food [129]. Reports have estimated high bat hunting and consumption in Sierra Leone, a major outbreak center [18], and Cameroon, a possible potent epicenter of future EBOV outbreaks [129]. Bushmeat consumption remains a primary source of nutrition for low-income families in many rural regions of Africa [130]. This can be partially linked to unrestrained fishing practices, leading to a decline in seafood sources, which results in an increased demand for bushmeat [131]. Additionally, bushmeat hunting is a traditional symbol of cultural identity promoted through generations [132,133]. Therefore, African populations have a high risk of contact with infected animals or their fluids during butchering and cooking.

Cultural reasons such as the traditional travel to a native village to die and be buried amongst ancestors, accompanied by unsafe funeral and burial practices (such as sleeping near a corpse or bathing in corpse wash water), are estimated to be responsible for 60–80% of the disease transmission [16,134]. The denial of traditional burial practices leads to resentment amongst locals, counter-productively increasing the practice and local beliefs [18,135].

In poverty-ridden equatorial African countries, the inadequate training of healthcare providers promotes nosocomial transmission [136,137]. A lack of precautionary measures and preparedness are significant contributors to transmission. In Guinea, for example, it took nearly 12 weeks for the 2013–2016 epidemic to be acknowledged, which delayed the prevention of the virus from spreading [138]. A shortage of trained clinicians, equipped laboratories, and emergency response teams further reduced the efficacy of the response [138]. Guinea, Liberia, and Sierra Leonne have only recently emerged from a long civil war, which resulted in the deterioration of education, transportation, and medical facilities. This could contribute to delayed communication and help outreach.

Epidemics resulting from the spread of vector-borne zoonotic pathogens often affect tropical rainforests and nearby African regions [139,140]. The diverse wild fauna in these rainforests could host deadly bacteria (such as *Salmonella* and *Campylobacter* species), viruses (such as HIV, Nipah, and herpes viruses), and parasites (such as *Nematoda* and *Toxoplasma gondii*) [141]. Epidemics caused by these pathogens represent a significant concern to already highly overwhelmed healthcare institutions. In addition, malnutrition and malaria (a common EVD co-infection) are rampant in the EBOV-endemic regions of Africa, contributing significantly to the disease progression and fatality rate [142,143].

Limited or a lack of education about the disease origin and transmission, cultural burial practices, and empathetic care for the ill contribute to ebolavirus spread. The rejection of “Western” medicines and attacks on HCWs have been reported on multiple occasions [144]. Deaths, even after the administration of trial drugs, have escalated the mistrust between HCWs and locals [145,146]. In the 2018 outbreak, local militias were involved in multiple attacks on HCWs, instigated by mistrust and misinformation [8]. Such incidences impede the efforts to control ebolavirus epidemics and provide adequate treatment [8]. Therefore, a high risk of another ebolavirus outbreak in Africa remains a possibility.

## 7. Current Status of Vaccines and Drugs

In the last two decades, multiple studies have been conducted to develop an ebolavirus vaccine and therapeutics [147,148,149,150,151,152,153,154,155,156,157,158,159,160,161,162,163,164]. In 2014, a randomized, double-blind, placebo-controlled, phase I clinical trial demonstrated the safety and immunogenicity of an ebolavirus vaccine [165]. An EBOV GP vaccine for prime-boost vaccination was developed in Russia in 2017 [166]. In clinical trials, this vaccine induced a humoral immune response with limited adverse effects [166]. Another vaccine was developed in China, where the ZEBOV GP was expressed using recombinant human adenovirus serotype 5 (Ad5) [167]. The Ervebo vaccine, containing the recombinant vesicular stomatitis virus expressing the EBOV glycoprotein (GP), received approval from the Food and Drug Administration (FDA) as the first licensed vaccine in the USA on 19 December 2019 [168,169]. REGN-EB3 (INMAZEB), a cocktail of three human monoclonal antibodies (atoltivimab, maftivimab, and odesivimab), was approved by the FDA on 14 October 2020 to be used in EBOV-infected adults and children [170]. Developed by Regeneron Pharmaceuticals, REGN-EB3 targets the EBOV GP [170]. This drug’s antiviral effect was linked to the stimulation of phagocytes and the inhibition of virus entry. Ansuvimab, also known as Ebanga or mAb114, is a human monoclonal antibody isolated from a patient in a 1995 outbreak [171]. This antibody binds to the LEIKKPDGS epitope in the GP1 subunit of the EBOV GP and prevents the virus from binding to the Niemann–Pick C1 receptor [171]. Developed by Ridgeback Biotherapeutics, Ansuvimab received FDA approval on 21 December 2020 to be used in EBOV-infected adults and children [171].

Despite these developments, the fight against ebolaviruses is obstructed by various limitations presented by different treatment platforms. These limitations include production difficulties and the high cost for virus-like particle (VLP)-based vaccines [172] as well as monoclonal antibody cocktails [173]; the requirement of regular booster dosages for DNA-based vaccines [174]; the low-efficacy of adenovirus-based vaccines (owing to pre-existing immunity) [175]; the low peptide stability and low immunogenicity of peptide-based vaccines [176]; the pre-screening requirement for blood-transmitted pathogens and the high toxicity of EVD convalescent plasma (CP) containing polyclonal antibodies [173,177,178]; and the possible absence of pan-ebolavirus protection in various treatment platforms [179]. These limitations indicate an urgent need for a safe, pan-ebolavirus global vaccine with limited side effects [180].

## 8. Future Challenges

There are several reasons for rapid EVD spread in endemic African countries: (a) a long lag period between the identification of an index case and the recognition of the outbreak [134,181]; (b) a delay in the emergency response by the monitoring authorities [182]; (c) insufficient HCW staffing, which is lower than one-fifth of the ratio recommended by the WHO (223 to 345 HCWs per 10^5^ people) [16,182]; (d) delayed international support due to the shortage of first-responder mobile laboratories [27,183]; (e) a dearth of dedicated aerial transport units and PPE for HCWs [27,183]; (f) inadequate protocols for the timely evacuation of HCWs and patients [27,183]; and (g) mistrust of the local community towards “Western” medicines, culminating in the form of attacks on HCWs [8,144]. In addition, the delayed implementation of ebolavirus testing in endemic sites, which are often located in remote and economically poor regions, contributes to virus spread during outbreaks [21,134,182]. Additionally, legal regulations in countries manufacturing antiviral drugs allow companies to regulate the medication price, making them less affordable for the local population [184]. Therefore, it would be significant to take effective administrative and legal measures to prevent the future shortage of medication during outbreaks.

**Figure 5:**
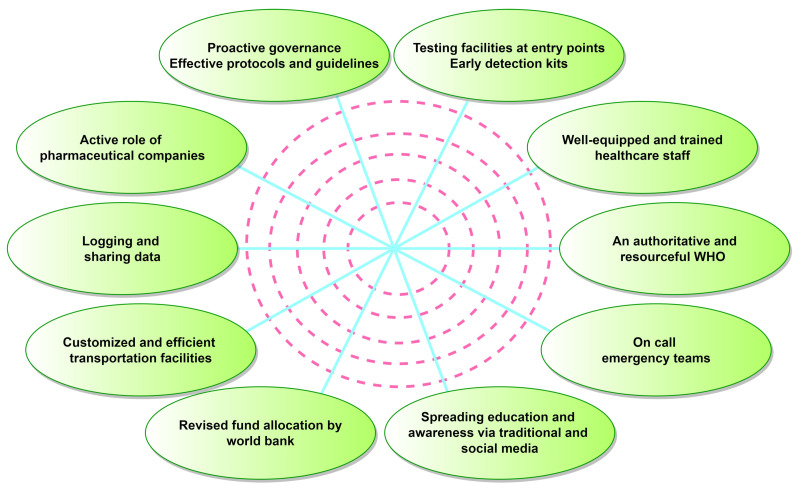
The ten tacks to thwart the global spread of ebolaviruses. The dotted circles represent communication and coordination channels at every level.

There is an urgent need to equip EVD testing facilities with rapid diagnostic kits with approved test protocols for early virus detection [185] (Figure 5). The early detection of a virus species causing an outbreak should be addressed [186]. Once the index case is identified, all contacts should be investigated for the rapid containment of the outbreak [181]. The importance of patient isolation, disinfection, proper ventilation, and barrier protection during patient and sample transportation needs to be emphasized [187,188,189]. During an outbreak, there is a need for coordination at different levels of government as well as healthcare control offices, transport authorities, non-governmental organizations (NGOs), military and emergency medical personnel, teams of scientists, laboratory workers, epidemiologists, and clinicians (Figure 5). Strictly regulated infectious disease surveillance systems and dedicated agencies supported by the government are needed to supervise the implementation of “all-parties-agreed” quarantine measures and to direct, control, and coordinate the prevention, containment, and relief efforts.

There is an urgent need for an ample stock of innovative PPE suitable for hot and humid climates in endemic countries [190,191,192]. Training courses and mock drills should be conducted to improve the skills of HCWs in donning, handling, and doffing PPE. Special attention should be paid to patient care training of the personnel [187]. The use of social media can help create EVD awareness amongst the local population, facilitating cooperation with HCWs. The involvement of local religious leaders would be significant, as they have an unprecedented authority within the local community [193]. Communication with local leaders could promote healthcare efforts to reduce the use of traditional burial practices and contact with hospitalized patients.

In this respect, the leading role of the WHO in the recruitment and training of healthcare and research personnel and volunteers is essential [16]. The World Bank’s contribution to future pandemic preparedness is needed for financial support in endemic regions of Africa (Figure 5). Additionally, the development of affordable drugs and vaccines by pharmaceutical companies is critical to prevent future epidemics. Measures should be taken to ensure the delivery of potent, experimental drug candidates to the affected areas [16].


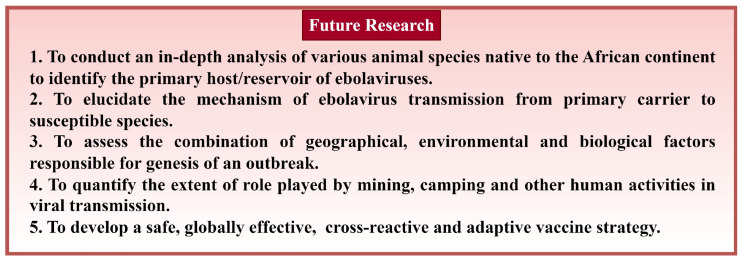


In the recent past, multiple epidemics and pandemics caused by zoonotic pathogens have been documented [139]. Therefore, proactive efforts such as the monitoring of wildlife diseases and “virus hunting” in wildlife are becoming essential to avoid animal-to-human transmission [194,195]. Such efforts can be complemented by mathematical models [196], simulation modeling approaches, and prediction algorithms, incorporating the data received from geographic information systems (GIS) as well as ecological, environmental, and wildlife surveillance departments [197]. The development of early diagnosis tests could lead to what we call “*prediction is better than cure*”. An excellent example of that statement is the successful prediction of an exponential increase in Rift Valley fever in Eastern Africa, facilitating an outbreak response and mitigation activities [198].

The strict adherence to the healthcare protocols in practice rather than in principle and coherent instead of fragmented action by authorities could prevent ebolaviruses from becoming pandemic viruses, ensuring protection from and the control of future outbreaks.

## Figures and Tables

**Table 3:** Total outbreaks, cases, and deaths caused by various ebolaviruses until 25 May 2022. We present the number of cases and deaths recorded by the CDC where possible. However, it should be noted that the actual number of confirmed cases might vary, as suggested by the ambiguity in various reports and published literature.

Ebolavirus	DRC		Gabon and RC		Uganda		South Sudan		West Africa		Total Number of Outbreaks	Total Deaths/Cases
	Outbreaks	Deaths/Cases	Outbreaks	Deaths/Cases	Outbreaks	Deaths/Cases	Outbreaks	Deaths/Cases	Outbreaks	Deaths/Cases		
**EBOV**	12	3173/4684	8	361/457	−	−	−	−	2	11,337/28,675	22	14,871/33,816
**SUDV**	−	−	−	−	4	235/449	3	180/335	−	−	7	415/784
**BDBV**	1	13/38	−	−	1	42/131	−	−	−	−	2	55/169
**TAFV**	−	−	−	−	−	−	−	−	1	0/1	1	0/1

## Data Availability

The datasets generated and analyzed during the current study are available from the corresponding author upon request.
